# Cardiac symptom attribution and knowledge of the symptoms of acute myocardial infarction: a systematic review

**DOI:** 10.1186/s12872-020-01714-8

**Published:** 2020-10-14

**Authors:** Benedikt Birnbach, Jens Höpner, Rafael Mikolajczyk

**Affiliations:** Institute of Medical Epidemiology, Biometrics and Informatics, Interdisciplinary Center for Health Sciences, Martin-Luther University Halle-Wittenberg, Halle (Saale), Germany

**Keywords:** Acute myocardial infarction, Acute coronary syndrome, Knowledge about symptoms, Symptom attribution, Awareness

## Abstract

**Background:**

Since the knowledge of the symptoms of acute myocardial infarction (AMI) may reduce the decision time for patients to seek help in case of an AMI, we aimed to summarize evidence on the knowledge of the AMI symptoms and the symptom attribution in case of an acute coronary syndrome (ACS).

**Methods:**

Therefore, we systematically searched the databases PubMed, CINAHL, Embase, and Cochrane Library for relevant studies published between January 1, 2008 and 2019 (last search August 1, 2019).

**Results:**

A total of 86 studies were included, with a composite sample size of 354,497 participants. The weighted mean of the knowledge scores for the symptoms of AMI of 14,420 participants from the general population, was 42.1% (when maximum score was considered 100%) and 69.5% for 7642 cardiac patients. There was a substantially better level of knowledge for six symptoms (‘chest pain or discomfort’, ‘shortness of breath’, ‘pain or discomfort in arms or shoulders’, ‘feeling weak, lightheaded, or faint’, ‘pain or discomfort in the jaw, neck, or back’, and ‘sweating’) (49.8–88.5%) compared to the four less obvious/atypical symptoms ‘stomach or abdominal discomfort’, ‘nausea or vomiting’, ‘headache’, and ‘feeling of anxiety’ (8.7–36.7%). Only 45.1% of 14,843 patients, who experienced ACS, have correctly attributed their symptoms to a cardiac cause.

**Conclusion:**

In conclusion, we found a moderate to good knowledge of “classic” and insufficient knowledge of less obvious symptoms of AMI. This might suggest that increasing knowledge about less obvious symptoms of AMI could be beneficial. It appears also important to address cardiac attribution of symptoms.

## Background

About 15.9 million acute myocardial infarctions (AMIs) occurred in 2015 and the aggregated number of AMIs has increased by 6.4% from 2005 to 2015 [1]. With an ageing population, and rising prevalence of obesity and diabetes in many countries, the prevention and therapy of cardiovascular disease will further increase in importance [1].

Since mortality or subsequent morbidity of AMI drastically decreases with a shorter time from symptoms-onset to reperfusion, [2–5] it is important to reduce any delays. One substantial component to ensure a timely treatment is patient delay, the time from symptoms-onset to seeking help. Here, attribution of symptoms to a cardiac cause has been found to be crucial [6–10]. In order to enable the patients to attribute the symptoms to the heart, knowledge of the symptoms of an AMI and the ability to recognize them seems to be beneficial [11–13]. However, to our knowledge, no review has attempted to summarize the findings on these factors systematically and give an overview of the world-wide knowledge levels of the AMI symptoms.

In our review, we present the current research status on AMI symptoms knowledge by systematically reviewing the literature and comparing the knowledge levels among the general population and cardiac patients. In addition, we report the cardiac symptom attribution among acute coronary syndrome (ACS) patients.

## Methods

### Screening process

**Fig. 1:**
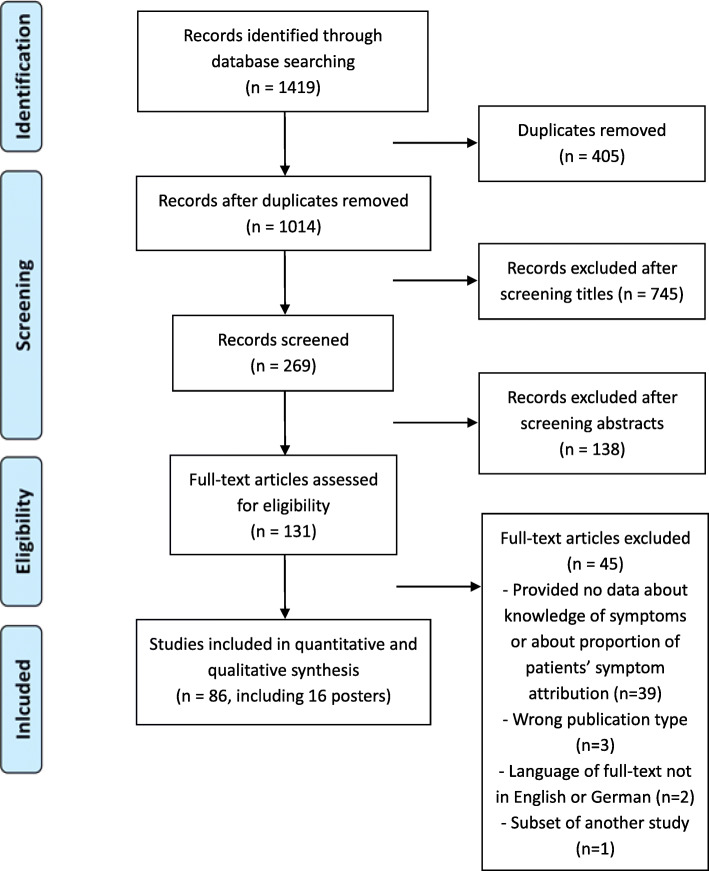
Screening process - flow chart based on Prisma guidelines [14]. The figure depicts the steps for finding the relevant literature: identification, screening, eligibility, and inclusion of literature

BB and JH conducted the literature search (Fig. 1) in the databases PubMed, CINAHL, Embase, and Cochrane Library and searched for publications from January 1, 2008 to 2019 (last search August 1, 2019). For PubMed, we used the search algorithm: “myocardial infarction”[mesh] AND (“chest pain” OR symptom* OR “warning signs”) AND (“recognition” OR “awareness” OR interpretation* OR perception* OR incongruence* OR congruence* OR expectation* OR “knowledge” OR “understanding” OR “community intervention” OR “educational intervention” OR campaign*) with the restrictions since 01/01/2008, languages: English or German, only human studies.

The search algorithms can be found in the Expanded methods section in the Additional file 1. We identified 1419 records, of which after deduplication 1014 remained for further analysis. After a further screening process by two reviewers (BB and JH), 86 publications were included in the analysis, of which 16 were conference abstracts or posters. Among them, 10 publications reported data from interventional studies.

### Inclusion criteria

The inclusion criteria were a sample size of more than 50 participants, date of publication after January 1, 2008, and English or German publication language. We included studies, if they offered data about the knowledge of the symptoms of AMI or data on the attribution of their ACS (AMI or unstable angina) to a cardiac or non-cardiac cause. Review articles, expert opinions or discussions, case reports, and letters were excluded.

### Extracted content

The criteria for the extracted content were set after initial scan of the publications, but before formal extraction started. The primary extraction was conducted by BB, while JH double-checked the extracted content. In case of discrepancies, BB and JH discussed the content and came to a consensus. The table that was used for extraction can be found in Additional file 2.

While there was a variety of methods how knowledge was investigated in these studies, in our review, we examined the knowledge levels (overall knowledge score) given by the studies (sometimes including trap questions and first responder questions), the knowledge level of chest pain as the most common symptom of AMI, [15] and of the 10 most frequently asked symptoms and one trap question. Trap questions were symptoms which usually do not occur in case of an AMI. First responder questions were items that asked about the appropriate response in case of an AMI, for example whether to call the ambulance.

Since previous studies have shown that recognizing symptoms yields higher knowledge scores than recalling them, we looked at open-ended and closed-ended questions separately [16, 17]. In order to compare the knowledge level in the general population and of cardiac patients, we established the following criteria for these groups:

1. General population: we included participants from studies which had as inclusion criteria an age range with a lower bound of 30 years or younger, and an upper bound of 60 years or older. In that way, we excluded studies which only included young or older participants. Additionally, the studies should not focus on a risk group, a certain ethnicity, or clinical staff, and should not include only one gender.
2. Cardiac group: we included samples of patients of any age that had a history of coronary artery disease (CAD) or a cardiac event, did not focus on a different risk group, and similarly did not focus on a certain ethnicity, on clinical staff, or only included one gender.

In this analysis, we only included conference abstracts or posters, when it was evident, whether open- or closed-ended questions were used. The data that we extracted from interventional studies was from the baseline data set or if not available, only extracted from the control group post intervention.

Finally, in order to evaluate the cardiac patients’ interpretation of their symptoms in case of an ACS, we assessed the symptom attribution in a ST-elevation myocardial infarction (STEMI) group, an ACS group that excluded the patients from the STEMI group, and a group including all cardiac patients.

### Quality assessment

BB and JH conducted a quality assessment based on the adapted version of the Newcastle Ottawa scale that has been used in previous studies to assess the quality of cross-sectional studies [18, 19]. Both authors assessed the studies, discussed the differences in assessment and finally unanimously agreed on a grading. The scale consisted of four questions about Selection (S1-S4), one question about Comparability (C1) and two questions about Outcome (O1, O2). Maximally 10 stars could be allocated. An overview of the allocated number of stars for each study can be found in Additional file 2 and a detailed report of the quality assessment in Additional file 3.

### Data analysis and reporting

For the purpose of comparing and summarizing the knowledge levels, we standardized the reported knowledge scores. The standardized score (the overall knowledge score in further text) is the mean knowledge score in the group, divided by the value that could maximally be achieved, multiplied by 100%. When calculating crude arithmetic mean, the overall knowledge score of each sample had the same weight. When calculating the weighted arithmetic mean, the weight of the overall knowledge score of each sample was based on the number of participants in each sample. For all analyses, we used SAS version 9.4.

## Results

### Study selection and quality assessment

Of the 86 included studies [9–11, 16, 20–101], 10 studies [44, 60, 72, 73, 75–77, 80, 81, 84] were interventional studies and could therefore not be rated with the adapted Newcastle-Ottawa-Scale. The remaining 76 studies were allocated a mean of 4.9 stars. Of those studies, 16 studies were conference abstracts which could only be assessed partly since some information about the studies was not included in the abstracts [30, 87–101]. Hence, only evaluating the available information the mean number of stars for these studies was 3.2. The 60 full-text articles that we assessed were allocated a mean of 5.4 stars. However, this relatively low number of stars can be explained by questions S4 and C1 which ask about the exposure and confounders. Since in 11 of those 60 studies the scientific question did not contain any exposure, and consequently no need for identifying confounders, those publications could be allocated no more than 6 stars instead of 10 (mean of the 11 studies: 3.0 out of 6 stars).

For our purpose only two criteria appeared relevant: representativeness of the sampling and response proportion. The representativeness was mostly rated high (mean: 0.9 out of 1 star), the response proportion was often high, too, but in only one study the comparability of responders and non-responders with respect to sociodemographic variables and clinical history was established.

### Study characteristics

The participants of the identified 86 studies were from 34 different countries. Several studies included samples from various countries, and we considered them as separate units (98 samples in total). A figure depicting the composition of countries can be found in the Additional file 1 (Additional figure 1). There were 26 samples from North America (27.1%), 25 from Europe (26.0%), 21 from East and South Asia (21.9%), 10 from Oceania (10.4%), nine from the Middle East (9.4%), and the remaining five from North/West Asia, South America, and West Africa (5.2%). Two conference abstracts did not specify where the samples were located.

Considering United Nations Human Development Index (HDI), [102] 69 samples (71.9%) were from 24 countries with a very high HDI, 19 samples (19.8%) from 10 countries with a high HDI, seven samples (7.3%) from three countries with a medium HDI, and one sample (1.0%) from a country with a low HDI.

The composite sample size of all studies was 354,497, excluding one study because information of sample size was not given [55]. The sample sizes ranged from 51 participants [81] to 76,864 [21] with a median of 400. Of all the included participants, 51.4% were male.

In the following analyses, the number of included studies varies as some outcomes were not reported by all studies.

### Overall knowledge score

#### Closed-ended questions

19 studies (27 samples) that used closed-ended questions reported a knowledge score. The studies designed their knowledge scores by offering a list to the participants with symptoms, and sometimes additionally with first responder questions or trap questions. The more symptoms the participants correctly identified and the more questions they answered correctly, the higher the score. Since the lists only had minor differences, we used the provided knowledge score of each study, standardized it to an overall knowledge score (dividing by maximum score value in the given study) and afterwards calculated the weighted mean and crude mean of all overall knowledge scores (see Methods, data analysis and reporting). The differences among the studies were that 11 samples were asked only about AMI symptoms, five additionally received one or more trap questions, one a first responder question, and two samples both, a first responder and a trap question. If the studies included participants that were not within the scope of the requirements for the population group or cardiac group that we defined above, we excluded the studies. Furthermore, we excluded studies that asked the patients what symptoms they had expected rather than asking for knowledge of symptoms. The weighted mean in the population group (42.1% of the maximal score) was substantially lower than in the cardiac group (69.5%) (Table 1).

**Table 1:** Overall knowledge score

Composition of sample, form of question	Number of samples	Number of countries	Participants	Crude mean [%] ^a^	Weighted mean [%] ^b^
Population group, closed-ended	10	10	14,420	36.5	42.1
Cardiac group, closed-ended	5	5	7642	54.7	69.5
Population group, open-ended	1	1	302	31.4	31.4
Cardiac group, open-ended	1	1	137	33.3	33.3

The table depicts the results by comparison of composition of sample and form of question used

^a^mean score in each study was standardized by dividing the mean by the maximum score; thus, the numbers report means in terms of percentage of maximum score

^b^as above; average mean was calculated from means from individual studies weighted by sample size

#### Open-ended questions

In the two studies that used open-ended questions, participants were asked about the symptoms, and the interviewer ticked off the items mentioned on an a priori defined list. The knowledge scores were lower than in most samples that were asked closed-ended questions, with 31.4% for the population group and 33.3% in the cardiac group.

### Chest pain knowledge

#### Closed-ended questions

With regard to the assessment of chest pain knowledge, when closed-ended questions were used, patients were given a list of symptoms that included chest pain and were asked: “Which of the following do you think is a symptom of a heart attack?” The phrasing of the question and description of the symptom differed in some studies. We included eight studies (16 samples) for the analysis of the population group and four studies (four samples) for the analysis of the cardiac group. Here, we also excluded studies that did not meet our requirements for population or cardiac group as defined above, or that asked about expectations rather than knowledge. Among both groups the knowledge of chest pain as symptom of AMI was similar (weighted means 88.2% vs. 86.2%) (Table 2).

**Table 2:** Knowledge of chest pain as symptom of acute myocardial infarction

Composition of sample, form of question	Number of samples	Number of countries	Participants	Crude mean [%] ^a^	Weighted mean [%] ^b^
Population group, closed-ended	16	14	145,631	83.5	88.2
Cardiac group, closed-ended	4	3	932	79.4	86.2
Population group, open-ended	4	4	7937	76.5	74.3
Cardiac group, open-ended	1	1	251	82.0	82.0

The table depicts the results by comparison of composition of sample and form of question used

^a^mean score in each study depicts the percentage of participants recognizing chest pain as symptom of acute myocardial infarction

^b^as above; average mean was calculated from means from individual studies weighted by sample size

#### Open-ended questions

When open-ended questions were used, the chest pain knowledge depicted the percentage of participants in each sample who reported chest pain, when asked to tell the interviewer about symptoms of a heart attack. For the analysis in the population group, we included four studies (four samples). We found a weighted mean (74.3%) that was smaller than in the group that was asked closed-ended questions. There was only one study including cardiac patients, which also reported a slightly smaller value (82.0%).

### Comparison of individual symptoms

When counting the different symptoms that the studies asked about and neglecting different phrasing or minor differences in the description of the symptoms, the studies assessed the knowledge of 26 different symptoms and 14 different trap symptoms.

In the following, we compared the 10 most frequently asked symptoms and the one most frequently asked trap symptom. For the purpose of description, we refer to a symptom as *moderately known,* if it is known by more than a third of all participants, and otherwise as *insufficiently known*.

#### Population group

In the population group, there were eight studies (16 samples) that applied closed-ended questions. Of those, two were convenience samples (studies 7, 8 in Table 3), and the remaining 14 samples were representative samples.

**Table 3:**
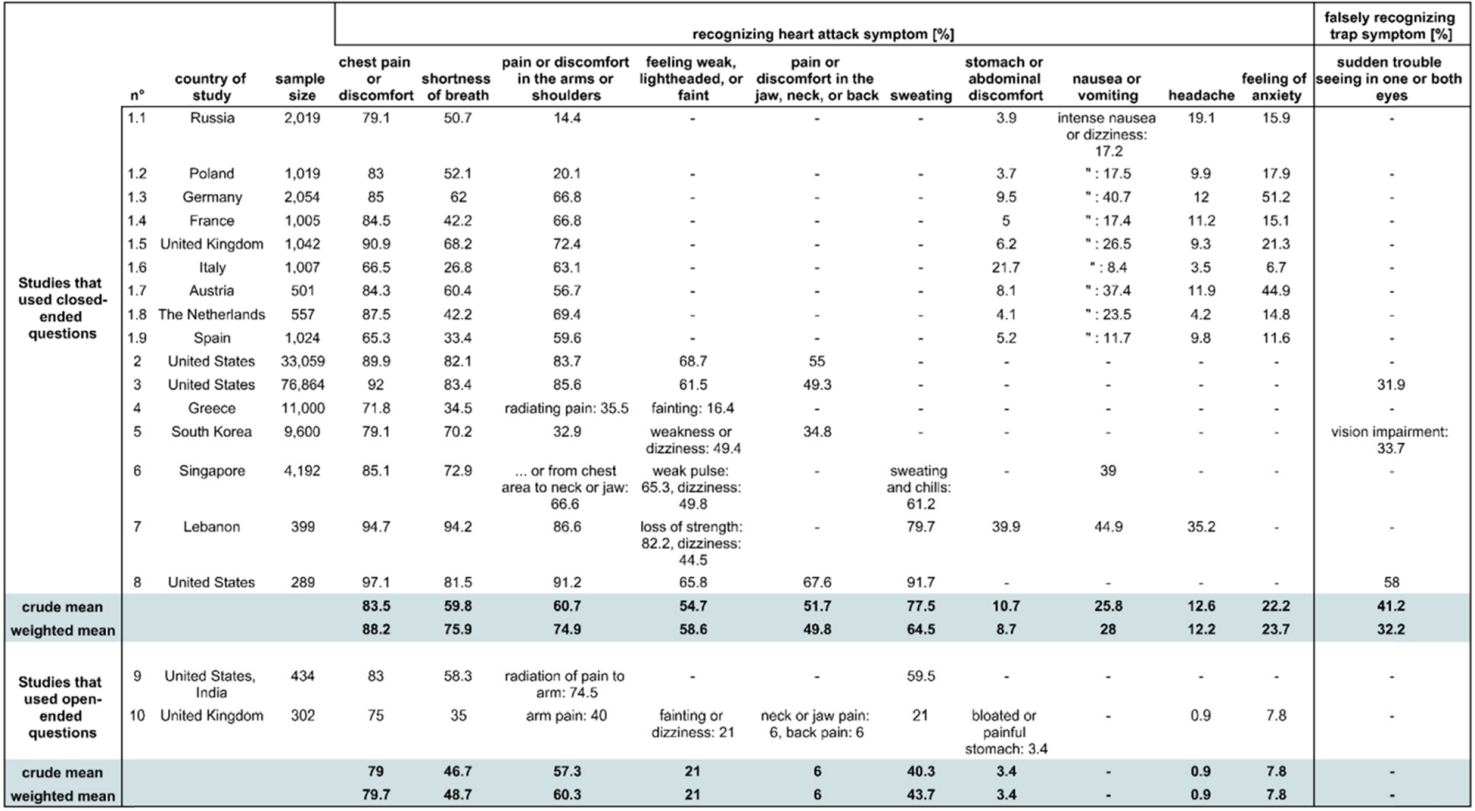
10 most frequently asked symptoms and one trap symptom in the population group

The table depicts crude mean and weighted mean for knowledge of symptoms of acute myocardial infarction in a group that was asked closed-ended questions and a group that was asked open-ended questions. If a study offered more than one knowledge percentage for a composite symptom, e.g. study 6 offered knowledge percentages on ‘weak pulse’ and ‘dizziness’ for the symptom ‘feeling weak, lightheaded, or faint’, we used the arithmetic mean of those percentages for our calculation. Studies included in the table: Study 1, [16] 2, [20], 3, [21] 4, [22] 5, [25] 6, [26] 7, [27] 8, [28] 9, [30] 10 [31].

##### Closed-ended questions

When closed-ended questions were used, the six symptoms ‘chest pain or discomfort’, ‘shortness of breath’, ‘pain or discomfort in arms or shoulders’, ‘feeling weak, lightheaded, or faint’, ‘pain or discomfort in the jaw, neck, or back’, and ‘sweating’ were *moderately known* (weighted mean: 49.8–88.2% of respondents who recognized the given symptom as a symptom of AMI) (Table 3). The mean of the weighted means of the *moderately known* symptoms was 68.7%.

Within the group of *moderately known* symptoms, only the symptoms ‘chest pain or discomfort’ and ‘sweating’ were known in all samples by more than half the participants. However, it should be noted that the symptom ‘sweating’ was only assessed in three samples. Furthermore, the four symptoms ‘stomach or abdominal discomfort’, ‘nausea or vomiting’, ‘headache’, and ‘feeling of anxiety’ were *insufficiently known* (weighted mean: 8.7–28.0%).

The weighted mean proportion of participants attributing the trap symptom ‘sudden trouble seeing in one or both eyes’ to AMI was 32.2% (Table 3), which suggests that there is confusion among some participants to differentiate between the symptoms of stroke and AMI. However, this trap symptom was only assessed in three samples.

##### Open-ended questions

When open-ended questions were asked, for every symptom, the weighted mean of the knowledge scores was lower than in the studies that used closed-ended questions. Since the two studies (two samples) which applied open-ended questions were convenience samples, we refrain from more detailed observations.

#### Cardiac group

In the cardiac group, two studies (two samples, studies 11 and 14 in Table 4) asked the patients what symptoms they had expected, and four studies (four samples, studies 12, 13, 15, 16 in Table 4) asked the patients what symptoms they recognized as AMI symptoms. Two studies (two samples, studies 17, 18 in Table 4) used open-ended questions to assess the patients' knowledge.

**Table 4:**
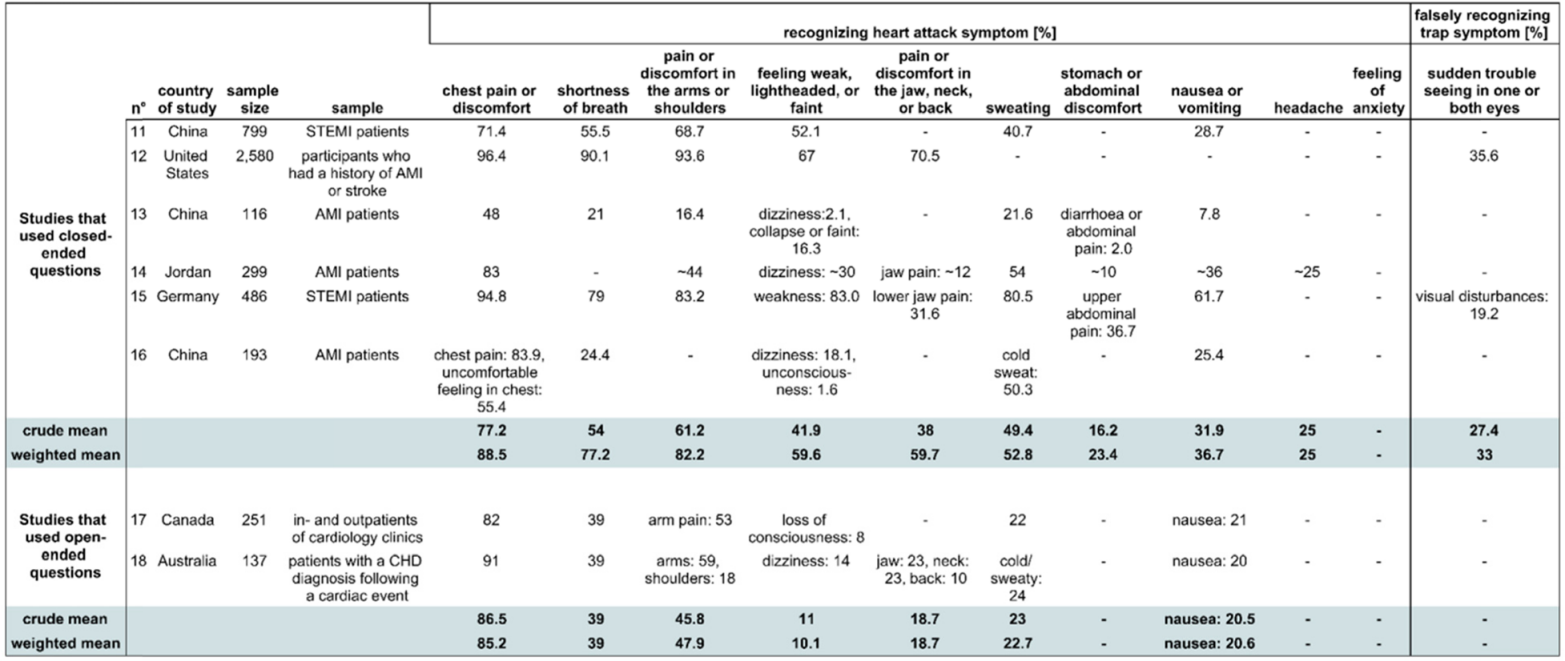
10 most frequently asked symptoms and one trap symptom in the cardiac group

The table depicts crude mean and weighted mean for knowledge of symptoms of acute myocardial infarction in a group that was asked closed-ended questions and a group that was asked open-ended questions. For our analysis, we proceeded as described in Table 3. STEMI = ST-elevation myocardial infarction. AMI = acute myocardial infarction. CHD = coronary heart disease. Studies included in the table: Study 11, [32] 12, [33] 13, [34] 14, [40] 15, [41] 16, [42] 17, [43] 18 [44].

##### Closed-ended questions

With regard to closed-ended questions, the same six symptoms that were *moderately known* in the population group were *moderately known* in the cardiac group (weighted mean: 52.8–88.5%). The mean of the weighted means of the *moderately known* symptoms was 70.0%. Regarding the four symptoms that were *insufficiently known* in the population group, there were no studies for the cardiac patient group that asked about ‘feeling of anxiety’ and only one study that asked about ‘headache’, in which about 25% of participants recognized it. Similar to the population group, the symptom ‘stomach or abdominal discomfort’ was also *insufficiently known* in the cardiac group with 23.4%. However, ‘nausea or vomiting’ was known by 36.7% in the cardiac group as opposed to 28.0% in the population group. Here, it is worth noting that an outlier in study 15 (Table 4) of 61.7% contributed to the higher result.

The trap symptom was only evaluated in two studies and the weighted mean proportion of an incorrect classification of this symptom was 33.0%.

##### Open-ended questions

In the two studies (two samples) that applied open-ended questions, similarly to the population group, the weighted mean for every symptom was smaller than when closed-ended questions were asked.

### Cardiac attribution

For calculating the proportion of ACS patients who attributed their symptoms to the heart, we analyzed 25 studies (25 samples) with 14,843 patients from 16 different countries. We looked at three groups, with some participants included in all three groups. Firstly, since a number of studies only included STEMI patients, we reported the cardiac attribution of a composite STEMI group. The STEMI group included patients from 11 studies from nine different countries with 4361 participants.

Secondly, in order to allow for a more representative depiction of the group of ACS patients, we reported the attribution of the remaining studies, excluding studies that only examined STEMI patients. To that group, 15 studies from 12 different countries with 11,442 patients contributed data, including one study with ACS patients, one study with patients about to be investigated for ACS, one study with patients with typical oppressive chest pain indicative for AMI, and the remaining 12 studies with AMI patients, of which two studies only included first-time AMI patients.

Thirdly, we reported the cardiac attribution of all studies.

The studies mostly evaluated the cardiac attribution by asking about the patients’ symptom attribution in general or specifically asking whether the patients attributed their symptoms to the heart. Some studies only looked at the symptoms-onset, by asking about the cardiac attribution of the initial symptoms. One study asked whether the reason to turn to a specialized service was that the patients believed the symptoms to be of cardiac origin [35]. The results indicate the percentage of patients who chose a cardiac interpretation.

In the STEMI group, the crude mean for cardiac attribution was 43.3% and the weighted mean 49.8%. In the group of ACS patients excluding the patients from the first group, the crude mean was 39.9% and the weighted mean 43.2%. All participants added up, their crude mean was 41.8% and their weighted mean 45.1%.

## Discussion

### Main findings

In our world-wide review, we found a moderate to good knowledge of “classic” symptoms of AMI and rather insufficient knowledge of less typical symptoms. Cardiac patients had substantially higher scores in a broader knowledge assessment compared to the general population. However, ‘chest pain’ as a lead symptom of AMI was equally known in the general population and among cardiac patients. We also found that less than half of patients attributed their symptoms to the heart.

### Knowledge of atypical symptoms

Our review showed that there is insufficient knowledge of atypical symptoms, which are especially relevant for women as they have a more atypical symptom presentation than men. While there is also considerable overlap among the symptoms men and women display, men present more often with the best known symptom ‘chest pain’ as well as ‘sweating’, which was among the best known symptoms in our comparison [103]. On the other hand, women present more often with *only* non-chest-pain discomfort, showing symptoms as ‘neck-, back- and jaw pain’ which was considerably less known, or with ‘nausea or vomiting’ which we found to be insufficiently known [15, 103–105]. Similarly, the observation is relevant for elderly people who also experience more atypical symptoms [5, 106, 107]. This lack of knowledge of atypical symptoms might be one factor for the higher patient delay and mortality among women and the elderly [108–110].

### Comparison of the population and cardiac patients

We found a higher knowledge in cardiac patients compared to the general population in a broader knowledge assessment (regarding the overall knowledge scores). When comparing the knowledge of each symptom separately, the knowledge of typical symptoms was similar among the groups, however cardiac patients had a higher knowledge of atypical symptoms. This suggests that the knowledge of atypical symptom might be the relevant difference.

The broader scope of knowledge in cardiac patients compared to the general population might be a consequence of their higher interest, the success of educational campaigns, or of counseling by their treating physician. It has to be considered as beneficial for delay time since previous studies observed that the time saving impact of knowledge in cardiac patients could mainly be attributed to knowledge of atypical symptoms [41]. One possible explanation for the beneficial effect is *symptom congruence,* [7] defined “as the extent to which one’s AMI symptom experience matches those expected of an AMI” [111]. By knowing more symptoms, including atypical symptoms, patients are more likely to choose a cardiac attribution and are not confused by the experience of unknown symptoms. Symptom congruence has been found to be beneficial for cardiac attribution and a higher cardiac attribution has been shown to be significantly associated with a shorter pre-hospital delay [7, 112–114].

### Knowledge and its relationship to cardiac attribution

However, in our review, despite a broad knowledge of symptoms, less than half of cardiac patients attributed their ACS (mostly AMI) to the heart when it happened.

This highlights the relevance of psychological factors. Knowledge alone is not sufficient for cardiac attribution, [9] and other components, for example various emotional factors also play a role [12, 115].

In fact, one study found that STEMI patients with a previous history of AMI or stent placement had a significantly lower knowledge score compared to those without it [41]. Albarqouni et al. pointed out that the driving factors might be “denial and psychological-trauma induced by the first attack” [41].

Strömbäck et al. showed that the appearance of atypical symptoms in a second AMI was not a predictor of a longer delay time [116]. One reason for this observation might be that a history of AMI seems to increase symptom congruence [111]. Therefore, the delay time in AMI survivors might not be primarily caused by a lack in knowledge but instead by psychological factors.

In the light of the above, it makes sense that despite our observed broad knowledge of symptoms in cardiac patients, a history of angina, AMI, or heart failure has not been found to have a positive impact on delay time. In fact, a history of angina or heart failure increases the delay time significantly in ACS [117].

All this highlights the significance of not merely educating patients about atypical symptoms in order to increase symptom congruence, but, maybe even more important, to prepare them psychologically. Cognitive and psychological factors that increase delay time have been observed to be denial, fear of troubling others, a lack of perceived seriousness of symptoms, a lack of perceived susceptibility to heart disease and a feeling of being able to cope with or control symptoms [118].

Therefore, we encourage future educational campaigns to not only focus their message on the knowledge of symptoms but also on overcoming psychological obstacles.

### Strengths, weaknesses, and sources of bias in the review

The strength of this review is the substantial number of studies from four databases. The weakness is that there are only few studies from Africa, Russia, and South America, as well as from countries with a medium or low HDI. Our review also potentially suffers from three sources of bias. First, the results of the studies might differ by the form of knowledge assessment chosen and the composition of the samples, especially where the participants came from. Secondly, since the compared cardiac groups and population groups did not include the same set of countries, these heterogeneities might have an effect on our analysis. Thirdly, there were inconsistencies among the studies whether the knowledge of some atypical symptoms like ‘headache’, ‘heartburn’, and ‘fever’ contributed positively to the overall knowledge score.

## Conclusion

We found a moderate to good knowledge of “classic” and rather insufficient knowledge of atypical symptoms of AMI. However, cardiac patients had a broader knowledge than the general population. As less than half of patients attributed their ACS to the heart when it happened, we see a potential to shorten delay time by educating about the symptoms, especially atypical symptoms, because they are common in the elderly and women, and because a broader knowledge increases symptom congruence. Furthermore, we encourage future campaigns to focus on overcoming psychological barriers that prevent patients from correctly identifying symptoms, attributing them to the heart, and reacting swiftly and appropriately.

## Supplementary information

**Additional file 1.**

**Additional file 2.**

**Additional file 3.**

## Data Availability

All data generated or analyzed during this review are included in this published article and its supplementary information files.

## Abbreviations

AMI: Acute myocardial infarction
ACS: Acute coronary syndrome
CAD: Coronary artery disease
STEMI: ST-elevation myocardial infarction
HDI: Human development index
