# Supplementary material for: Cardiac symptom attribution and knowledge of the symptoms of acute myocardial infarction: a systematic review
Source: BMC Cardiovasc Disord. 2020 Oct 14;20:445. doi: 10.1186/s12872-020-01714-8 (PMC7557019; doi:10.1186/s12872-020-01714-8)
Supplement: Supplementary file 1 — Additional file 1. [file 12872_2020_1714_MOESM1_ESM.pdf]

SUPPLEMENTAL MATERIAL

Expanded Methods

Search algorithms used for literature search:

PubMed:

“myocardial infarction”[mesh] AND (“chest pain” OR symptom* OR “warning signs”) AND (“recognition” OR “awareness" OR interpretation* OR perception* OR incongruence* OR congruence* OR expectation* OR "knowledge" OR “understanding” OR “community intervention” OR “educational intervention” OR campaign*)

- ➔ Restrictions: Since 01/01/2008, in English or German, studies on humans

CINAHL:

(MW myocardial infarction) AND (TX chest pain OR TX symptom* OR TX warning sign*) AND (TX recognition OR TX awareness OR TX interpretation* OR TX perception* OR TX incongruence* OR TX congruence* OR TX expectation* OR TX knowledge OR TX understanding OR TX community intervention* OR TX educational intervention* OR TX campaign*)

- ➔ Restrictions: Since 2008, in English or German, studies on humans

EMBASE:

'heart infarction'/exp/mj AND ('chest pain':ab,ti OR 'symptom*':ab,ti OR 'warning sign*':ab,ti) AND ('recognition':ab,ti OR 'awareness':ab,ti OR 'interpretation*':ab,ti OR 'perception*':ab,ti OR 'incongruence*':ab,ti OR 'congruence*':ab,ti OR 'expectation*':ab,ti OR 'knowledge':ab,ti OR 'understanding':ab,ti OR 'community intervention*':ab,ti OR 'educational intervention*':ab,ti OR 'campaign*':ab,ti) AND [2008-2019]/py AND ([english]/lim OR [german]/lim) AND [humans]/lim

Cochrane Library:

#1: MeSH descriptor: [Myocardial Infarction] explode all trees

#2: ("chest pain" or symptom* or "warning signs") and ("recognition" or "awareness" or interpretation* or perception* or incongruence* or congruence* or expectation* or "knowledge" or "understanding" or "community intervention" or "educational intervention" or campaign*)

#3: #1 and #2

➔ Restriction: Since 2008

Supplemental Figures and Figure Legends

Additional Figure 1. Frequencies of countries.

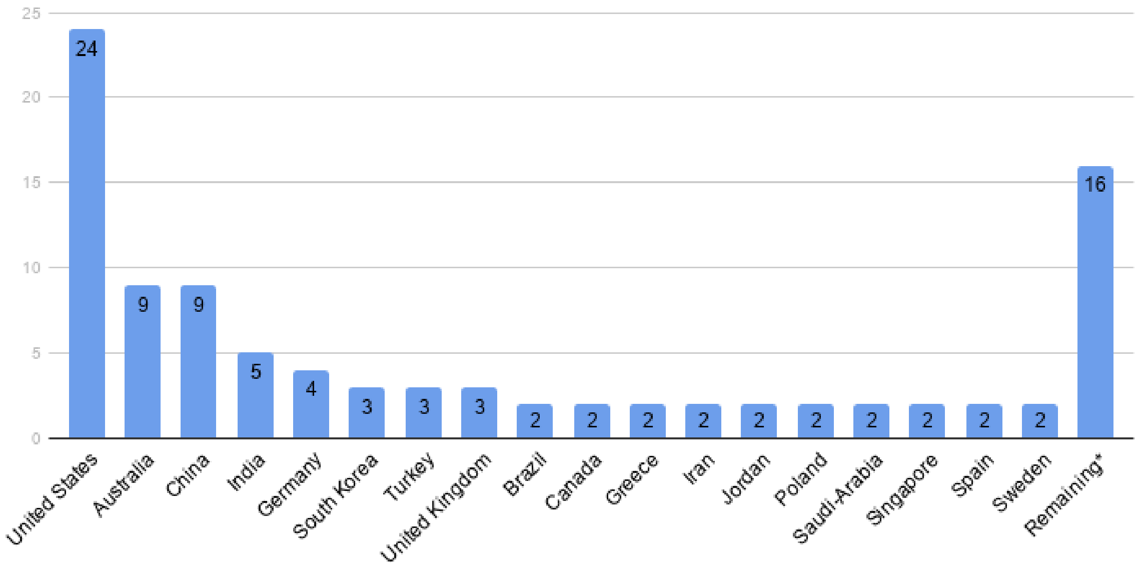

Bar chart with number of samples from each country on y-axis. When a study took place in more than one country, we counted one sample for each country where the participants were located. *The remaining countries have one sample each.
